# Exploring the role of gaze behavior and object detection in scene understanding

**DOI:** 10.3389/fpsyg.2013.00917

**Published:** 2013-12-06

**Authors:** Kiwon Yun, Yifan Peng, Dimitris Samaras, Gregory J. Zelinsky, Tamara L. Berg

**Affiliations:** ^1^Computer Science Department, Stony Brook UniversityStony Brook, NY, USA; ^2^Psychology Department, Stony Brook UniversityStony Brook, NY, USA; ^3^Department of Computer Science, University of North Carolina, Chapel-HillChapel Hill, NC, USA

**Keywords:** gaze, computer vision, description, scene understanding, object detection, image annotation

## Abstract

We posit that a person's gaze behavior while freely viewing a scene contains an abundance of information, not only about their intent and what they consider to be important in the scene, but also about the scene's content. Experiments are reported, using two popular image datasets from computer vision, that explore the relationship between the fixations that people make during scene viewing, how they describe the scene, and automatic detection predictions of object categories in the scene. From these exploratory analyses, we then combine human behavior with the outputs of current visual recognition methods to build prototype human-in-the-loop applications for gaze-enabled object detection and scene annotation.

## 1. Introduction

Every day we consume a deluge of visual information, either by looking at images and video on the web, or more generally by looking at the visual world unfolding around us. For the first time in history, this viewing behavior is accompanied by a rapidly increasing number of cameras turned toward the user that could conceivably *watch us back*. Whether it is webcams on laptops, front-facing cell phone cameras, or Google Glass, the media that we use to access imagery increasingly has the potential to observe our viewing behavior. This provides an unprecedented opportunity to harness these devices and to make use of eye, head, and body movements for human-in-the-loop intelligent systems. In particular, cues from our behavior during natural viewing of imagery provide information regarding the content we find important and the tasks that we are trying to perform. This is particularly true in the case of gaze behavior, which provides direct insight into a person's interests and intent.

We envision a day when reliable eye tracking can be performed using standard front facing cameras, making it possible for visual imagery to be tagged with individualized interpretations of content, each a unique “story,” simply through the act of a person viewing their favorite images and videos. In this paper we provide a glimpse into this exciting future by analyzing how humans interact with visual imagery in the context of scene memory and scene description tasks, and propose a system that exploits this behavior to assist in the detection (or tagging/annotation) of objects that people find important. We see the potential for a symbiotic relationship between computer scientists and behavioral scientists to advance understanding of how humans interact with visual data and to construct new human-computer collaborative systems for automatic recognition of image content. For computer scientists, understanding how humans view and interpret images will lead to new methods of designing, training, evaluating, and perhaps augmenting computer vision systems for improved image understanding. For behavioral scientists, these methods from computer vision will inform the relationship between viewing behavior and image content that will be essential to building comprehensive theories and models of scene understanding.

### 1.1. Visual recognition and detection

**Figure 1:**
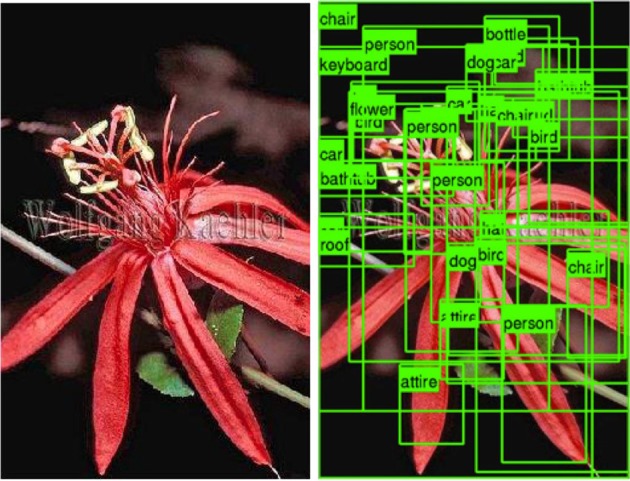
**Detection results for 120 common object categories**.

In computer vision, visual recognition algorithms are making significant progress. Recent advances are enabling recognition problems to be approached at a human scale, classifying or localizing thousands of object categories with reasonable accuracy (Deng et al., 2010, 2012; Lin et al., 2011; Krizhevsky et al., 2012; Perronnin et al., 2012). However, despite rapid advances in methods for detecting and recognizing objects in images (Deng et al., 2012; Everingham et al., 2012), they are still far from perfect. As evidenced in Figure 1, running 120 common object detectors on an image produces unsatisfactory results. Detectors still make noisy predictions. In addition, even if the detectors were completely accurate, they would produce an indiscriminate labeling of *all* objects in an image. Although knowing all of the objects in a scene would be useful for some applications, it would probably not make a good model for human scene understanding. Scene understanding, at its minimum, must reflect the fact that some objects are interpreted as being more important than others, and that different viewers may place different levels of importance on these objects. Current computer vision approaches therefore treat scene understanding as image understanding, attaching equal importance to every object that can be recognized in a pattern of pixels. This is probably not how people understand scenes and might not be the right output for end-user applications which by definition should be guided by human interpretations.

### 1.2. Information from gaze

It has long been known that eye movements are not directly determined by an image, but are also influenced by task Yarbus (1967). The clearest examples of this come from the extensive literature on eye movements during visual search (Neider and Zelinsky, 2006a; Zelinsky, 2008; Judd et al., 2009; Zelinsky and Schmidt, 2009); specifying different targets yields different patterns of eye movements despite the image remaining the same (the same pixels). However, clear relationships also exist between the properties of an image and the eye movements that people make during free viewing. For example, when presented with a complex scene, people overwhelmingly choose to direct their initial fixations toward the center of the image, probably in an attempt to maximize the extraction of information from the scene (Renninger et al., 2007). Figure/ground relationships play a role as well; people prefer to look at objects even when the background is made more relevant to the task (Neider and Zelinsky, 2006b). All things being equal, eye movements also tend to be directed to corners and regions of high feature density (Mackworth and Morandi, 1967; Tatler et al., 2006), sudden onsets (Theeuwes, 1994; Theeuwes et al., 1999), object motion (Itti, 2005; Itti and Baldi, 2009), and regions of brightness, texture, and color contrast (Itti and Koch, 2000, 2001; Parkhurst et al., 2002). These latter influences can all be considered saliency factors affecting object importance. Behavioral research has therefore provided a wealth of information about the objects in a scene that people find important, and how this is affected by the properties of these objects, but very little is known about how one's ability to detect these objects factors into their scene understanding.

Rather than focusing on object salience, in our experiments we ask: how categories of objects or events, and their detectability, might influence gaze [see also, Einhäuser et al. (2008)], and how we can use gaze to predict semantic categories. Eye movements can inform image understanding in two different but complementary ways. First, they can be used to indicate the relative importance of content in an image by providing a direct measure of how a person's attention was spatially and temporally distributed. Second, the patterns of saccades and fixations made during image viewing might be used as a direct indication of content information. For example, to the extent that gaze is drawn to oddities and inconsistencies in a scene (Tatler, 2007), fixations might be used to predict unusual events (Baldi and Itti, 2010).

### 1.3. Human-computer collaboration

**Figure 2:**
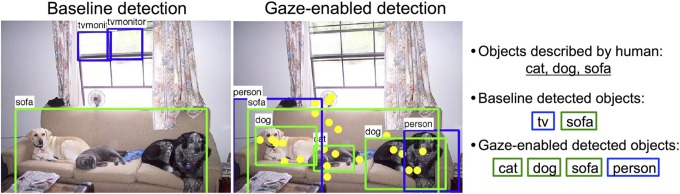
**Left:** Baseline detection results using 20 deformable part models from Felzenszwalb et al. (2010) with default thresholds including correct detections (green) and incorrect detections (blue). **Middle:** Gaze-enabled detection results with fixations (yellow). **Right:** Objects described by people and detected objects from each method (green - correct, blue - incorrect).

In this paper we explore the potential for combining behavioral and computational inputs into integrated collaborative systems for image understanding. There are many recognition tasks that could benefit from gaze information, with the prototype system for human-computer collaborative image classification by De Campos et al. (2012) being just one example. In this paper we focus on object detection and annotation. Figure 2 suggests the potential benefits of such a human-computer collaborative object detection system. Rather than applying object detectors at every location in an image arbitrarily, they could be more intelligently applied only at important locations, as indicated by gaze fixations. This would not only minimize the potential for false positives, but also constrain the true positives to only the content considered most important by the viewer. It might also have implications for efficiency in real-time detection scenarios.

Central to making these systems work is our belief that humans and computers provide complimentary sources of information for interpreting the content of images.

Humans can provide:

- Passive indications of content through *gaze* patterns. These cues provide estimates about “where” important things are, but not “what” they are.
- Active indications of content through *descriptions*. These cues indicate “what” content in an image is important to a viewer, but may give only a rough idea of “where” this content is located.

Computer vision recognition algorithms can provide:

- Automatic indications of content from recognition algorithms; estimates of “what” might be “where” in visual imagery. But, these predictions will be noisy and will lack knowledge of relative content importance.
- Computer vision can piece together these various cues as to “what” important content is located “where” in a scene.

Human-Computer collaborative systems have the potential to make great strides in semi-automated scene understanding, or image understanding, as it is referred to in the computer vision community. It is our position that image understanding is ultimately a human interpretation, making it essential that inputs from humans be integrated with computational recognition methods. Attempts to solve this problem through analysis of pixels alone are unlikely to produce the kind of image understanding that is useful to humans, the ultimate consumers of imagery. However, in order to build such a collaborative system we first have to better understand the relationship between these various “what” and “where” cues.

In this study, we describe several combined behavioral-computational analyses aimed at exploring the relationships between the pixels in an image, the eye movements that people make while viewing that image, and the words that they produce when asked to describe it. To the extent that stable relationships can be discovered and quantified, this will serve the synergistic goals of informing image interpretation algorithms and applications, and contribute to the basic scientific knowledge about how people view and interpret visual imagery. For these analyses we collected gaze fixations and image descriptions from two commonly used computer vision datasets. Our data, the SBU Gaze-Detection-Description Dataset, is available at http://www.cs.stonybrook.edu/~ial/gaze.html.

## 2. Dataset and methods

We conducted two sets of experiments, using two commonly explored computer vision image datasets with varying task scenarios, designed to address slightly different questions. We will refer to these experiments by their respective dataset name, the Pascal VOC dataset (Everingham et al., 2008) and the SUN 2009 dataset (Choi et al., 2010).

### 2.1. Pascal VOC

**Figure 3:**
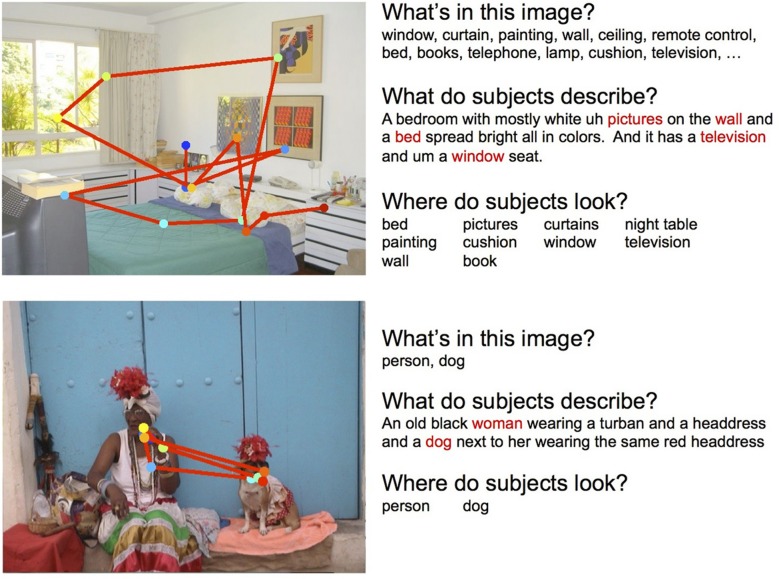
**Examples of scanpaths and descriptions for the SUN09 (top) and Pascal (bottom) experiments. Left:** Gaze patterns where each dot indicates a fixation. Colors indicate earlier (blue) to later (red) fixations. **Right:** Descriptions of the images, together with the object ground truth and the objects that were fixated. Red words indicate objects automatically extracted from the text description.

The goal of this experiment was to determine the relationships between object detections and gaze fixations, and object detections and scene descriptions, for a large number of images. The Pascal VOC dataset is well suited to this purpose. The Pascal VOC is a visual recognition challenge widely known in the computer vision community where models of object category detection (among other tasks) are evaluated. We used the 1000 images selected by Rashtchian et al. (2010) from the 2008 dataset (Everingham et al., 2008), which included at least 50 images depicting each of 20 object categories. Deformable-part model object detectors for each of these categories were provided by Felzenszwalb et al. (2010). These 1000 images were then shown to three participants (experimentally naïve and with normal or corrected to normal vision), who were instructed to freely view the scenes in anticipation of a memory test; after every 20 image presentations, a test image would appear and the participant would have to indicate whether it was shown previously (50% yes, 50% no). Each image was presented for 3 s and subtended 18 degrees of visual angle. Eye position was sampled at 1000 Hz using an EL1000 eye tracker (SR Research) with default saccade detection settings. Descriptions of each scene were obtained using Amazon's Mechanical Turk (AMT), provided by Rashtchian et al. (2010), and generally captured image content relating to objects (“man”), relationships between objects (“man drinking a beer”), and occasionally the scene category name (“man drinking a beer in a kitchen”). Given the goals of the experiment, note that scene descriptions were not obtained from the behavioral participants, as this would have required a prohibitively long time for 1000 images. Instead, this allowed us to determine gaze and description patterns that hold across different people—something that would be necessary in the context of end-user applications such as image search. Figure 3 (bottom) shows a sample scanpath and a AMT provided description for one scene.

### 2.2. SUN09

The goal of this experiment was to determine the three-way relationships between object detections, gaze, and scene descriptions, for a modest number of more complex images. The SUN 09 dataset (Choi et al., 2010) is well suited to this purpose, as the images tend to be more cluttered compared to Pascal (which typically depict only a small number of objects). We selected 104 images of 8 scene categories, each of which had hand-labeled object segmentations. We then trained 22 deformable-part model object detectors (Felzenszwalb et al., 2010) using images with associated bounding boxes from ImageNet (Deng et al., 2009). These object detectors were selected to cover, as much as possible, the main object content of our selected scenes. Each of these scenes, subtending 29 degrees of visual angle (1280 x 960 pixels) were presented for 5 s to eight participants, who were asked to perform a scene description task. Immediately following each presentation, participants were given 20 s to verbally describe the previously viewed scene. These descriptions were recorded and later transcribed to text. Additional text descriptions were also obtained using AMT following the procedure outlined in Rashtchian et al. (2010). Eye movements were recorded throughout scene viewing using the same eye tracker and saccade detection settings. Figure 3 (top) shows a sample scanpath and description for one scene from one participant.

## 3. Results

In this section we address several general relationships between fixation, description, and image content: (1) What do people look at? (Section 3.1), (2) Do people look at the same things? (Section 3.2), (3) What do people describe? (Section 3.3), and (4) What is the relationship between what people look at and what they describe? (Section 3.4).

### 3.1. What do people look at?

#### 3.1.1. Fixations on selected objects

All analyses will be restricted to those selected object categories from the image datasets for which we have trained detectors. For the Pascal dataset, the 20 selected categories constitute the entire set of object categories available for the 1000 images used in our study. However, there are an unknown number of other objects depicted in these images for which no object segmentations or detectors are available. For the SUN09 dataset, the 22 object categories that we selected represent only a subset of the total number objects appearing in our 104 images, many of which were densely labeled (almost every pixel was associated with an object). However, many of these objects were very small or infrequent categories (e.g., a television remote control) or corresponded to background regions (e.g., walls, ceilings). On average, the number of objects per image from the SUN09 ground truth was 32.8, 46.1% of which were objects from our selected categories. More importantly, we found that 76.3 and 65.6% of fixations made during scene viewing fell within bounding boxes surrounding the selected object categories for the Pascal and SUN09 datasets, respectively. Therefore, while we are not able to make conclusions about all of the objects appearing in these images, the objects that we are able to evaluate seem to capture the content of these scenes that participants found most interesting.

#### 3.1.2. Fixations vs. object type

What types of objects in a scene most strongly attracted a person's attention? We estimated this by computing a normalized proportion of fixations on objects, where objects of different types are normalized for size differences (larger objects might be fixated more frequently simply by chance). More specifically, given an image, a normalized proportion of fixations for each object type is defined as, *NF*(*I*, *b*):

(1) $$\;F(I,b)=\frac{\#\text{fixations in bounding box }b}{\#\text{fixations in image }I}$$

(2) $$\;B(I,b)=\frac{\text{size of bounding box }b}{\text{size of image }I}$$

(3) $$NF(I,b)=\frac{F(I,b)}{B(I,b)}$$

where *F* (*I*, *b*) indicates the proportion of fixations falling into bounding box *b* in image *I*, and *B*(*I*, *b*) indicates the ratio of the size of bounding box *b* to the whole image. *NF*(*I*, *b*) denotes the normalized proportion of fixations of bounding box *b* in image *I*.

**Figure 4:**
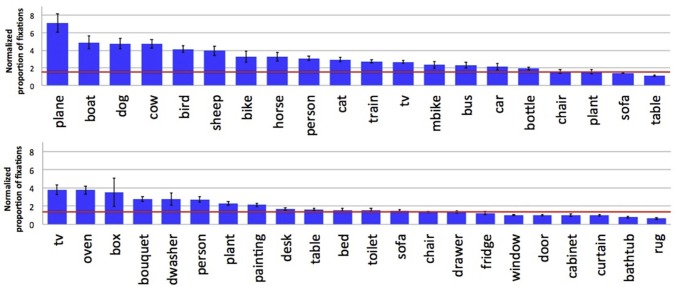
**Normalized proportion of fixations by category for the Pascal (top) and SUN09 (bottom) datasets**. A baseline indicating chance fixation was computed for individual participants by finding for each trial another trial with the same number of fixations, but for a different scene. The red lines indicate the average of these by-subject baselines. By doing this, factors such as central fixation biases and individual scanning strategies are partially controlled.

Figure 4 shows results for each object category from the two datasets. For the Pascal dataset people tend to look preferentially at animals and people, and at less typical vehicles (planes and boats) relative to common household items like tables, chairs, and potted plants. For the SUN09 dataset, which depicted far fewer animals, people were more likely to look at other people, and content elements like televisions (when they were on), ovens, and boxes. Collapsing across the two datasets, we found that more fixations were devoted to inspecting animate objects (*P*(fixated | present) = 0.636) compared to inanimate categories (*P*(fixated | present) = 0.495).

**Figure 5:**
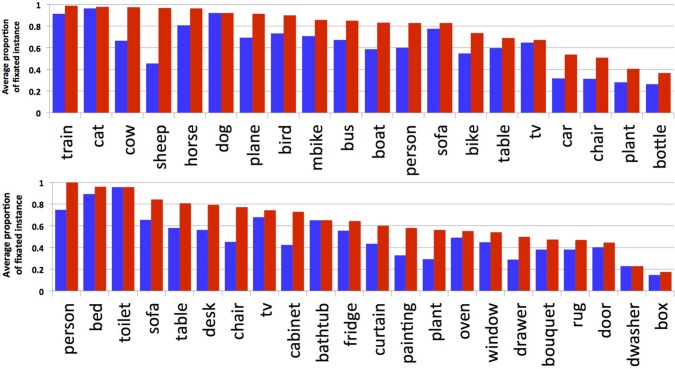
**Proportion of fixated categories**. Blue bars show the average proportion of fixated instances per category. Red bars show the percentage of images where a category was fixated when present (at least one fixated instance in an image).

Figure 5 shows the probability of an object category being fixated, grouped by whether at least one instance of an object type in the scene was fixated (red bars) or whether each instance of the object was fixated (blue bars; measured as the average proportion of fixated instances for a given object type). This distinction is important when evaluating the relationship between fixations and objects in scene understanding; whereas the fact that sheep are present in a scene is likely to be important for understanding the scene, resulting in a fixation on a sheep, it is probably unnecessary to fixate on every sheep instance in order to interpret the scene's content. Our analysis would seem to bear this out. Continuing with the sheep example, whereas only 45% of all sheep were fixated, at least one sheep was fixated in 97% of the images containing sheep. The other categories show a similar pattern, although not as extreme in difference. Note that categories showing no difference between these measures, such as dishwasher and toilet, reflect the fact that only one instance of these categories appeared in the scene, and not the fact that each of multiple instances was fixated. Note also, that object categories such as person, cat, and dog are nearly always fixated at least once when they are present, but inanimate and common objects such as bottles and potted plants are fixated much less frequently. Collectively, we interpret these data as suggesting that some objects are more important than others for scene understanding, although it is typically unnecessary to fixate every instance of even the important objects.

#### 3.1.3. Locations of fixations on objects

**Figure 6:**
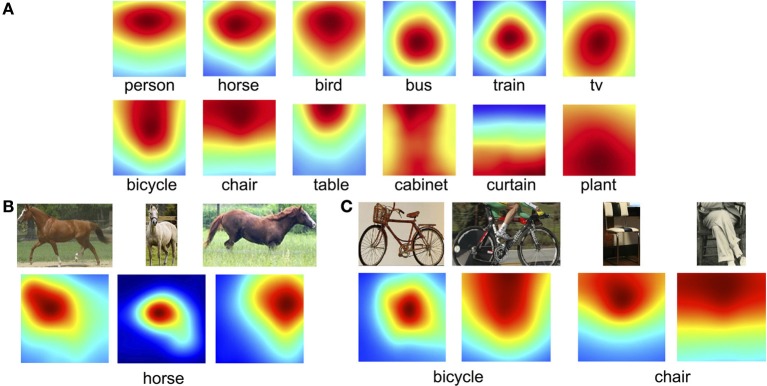
**Average fixation density maps for select object categories. (A)** Examples showing the category dependent nature of fixation patterns. **(B)** Representative samples of a face fixation bias, shown here for horses. **(C)** A bias to fixate the tops of bicycles was driven by cases showing a rider, but the tops of chairs and tables were fixated regardless of the depiction of a person.

The previous analyses focused on what object categories were fixated. Here we analyze where these fixations tended to land on objects, and how these preferred viewing positions tended to vary by object type. To do this, we first computed a fixation density map over the object bounding box using a two-dimensional Gaussian distribution with a sigma chosen for each image to approximate a one-degree fovea. Gaussians were summed to obtain fixation density maps, and these were then averaged over all the bounding boxes for a given object category. Figure 6A shows some particularly interesting examples of these averaged object-based fixation density maps. Consistent with previous work (Nuthmann and Henderson, 2010), fixations on some objects, such as buses and trains and televisions, tended to cluster on the centers of the objects. However, for other objects, such as plants, this distribution was far more uniform, as was the case for objects occupying a large percentage of the image, such as cabinets and curtains [but see Pajak and Nuthmann (2013)]. Moreover, we found that fixations on animals (people, horses, birds) tended to be skewed to the top of the bounding boxes, reflecting the fact that people tend to look at faces during scene viewing. We verified this observation by dividing these animal categories based on the where the face appeared in image and found that fixation preference indeed followed the location of the face (Figure 6B). Interestingly, similar fixation biases were found for objects that have strong functional associations that place differential importance on parts of the objects. For example, fixations were clustered towards the tops of bicycles, chairs and tables (Figure 6A), perhaps because people often sit on the tops of chairs and the seats of bicycles, and place things on the tops of tables. This observation, however, is potentially confounded by the fact that some of the images depicted people sitting on bicycles and chairs, thereby making it unclear whether fixations were biased toward the tops of these objects or rather the centers of the bicycle+person or chair+person objects. To tease apart these possibilities we again divided images into those showing isolated bicycle/chair/table objects and those showing these objects with a person sitting on them (Figure 6C). We found that, whereas subjects did tend to look at the center of bicycles and motorbikes when they did not also depict a rider, subjects preferred to fixate the tops of chairs and tables even when these objects appeared without a person. This analysis therefore partially supports previous work showing that fixations tend to land on the centers of objects, but qualifies this work by showing that this center-fixation bias does not hold for objects that typically interact with another object, such as people sitting on chairs, and boxes and magazines sitting on tables. This analysis also suggests that where people tend to distribute their fixations on a particular type of object may provide information that can assist in the recognition of that object class.

We also analyzed the relationship between gaze, bounding boxes, and the true object segmentations from the SUN09 dataset. We did this by computing the percentages of the bounding box areas that overlapped with the actual object segmentations (a measure of bounding box—segmentation difference), and comparing these to the percentages of fixations in the bounding boxes that also fell on the segmented objects. These results are shown in Table 1 for all of the selected SUN09 categories, and some representative examples. We found that the mean percentage of fixations on the actual object segmentations were similar to the mean percentage of bounding box area occupied by the segmented objects. This suggests that distributions of fixations, while potentially useful in indicating the category of object being fixated, will probably not be useful in refining bounding box predictions to object segmentations.

**Table 1:** **Comparison between segmentations and bounding boxes**.

	**All**	**Person**	**Chair**	**Painting**
% of area	68.41	52.74	57.51	91.09
% of fixations	68.97	58.84	59.14	91.47

Reported is the percentage of the bounding box area occupied by the segmented object, and the percentage of the bounding box fixations that fell on the segmented object.

### 3.2. Do people look at the same things?

To analyze the consistency of fixations across participants, we adopted the agreement score used in Judd et al. (2011). Using the leave-one-out procedure, we first created a fixation density map averaged over *n*-1 of the participants, then used this map to predict the fixations of the participant who was left out. Fixation density maps were computed using a Gaussian, with a sigma corresponding to one degree of visual angle, centered on each fixation and weighted by fixation duration. An agreement score was computed in terms of area under the curve (AUC) using the procedure described in Judd et al. (2011). These scores ranged from 0.5, indicating chance agreement, to 1, indicating perfect agreement.

**Figure 7:**
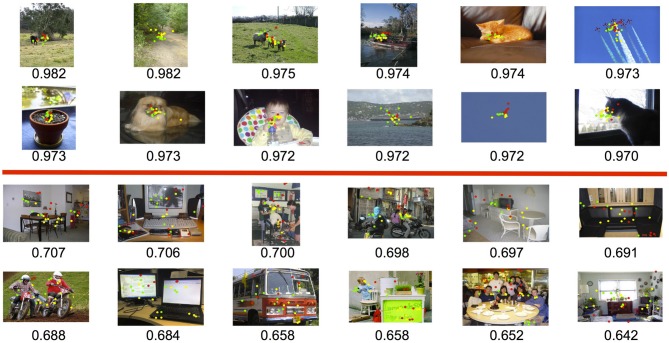
**Examples of high (top) and low (bottom) fixation agreement among participants using the Pascal dataset**. The red, green, and yellow dots indicate fixations from our three participants.

**Figure 8:**
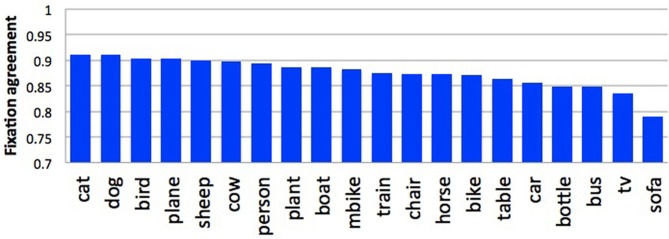
**Fixation agreement by object type for the Pascal dataset**.

Figure 7 shows some examples of high-agreement scenes (top) and low-agreement scenes (bottom) from the Pascal dataset, with their corresponding agreement scores. In general, we found that agreement in participant's fixation behavior tended to decrease with the number of objects in the scene (*r* = −0.755). This was to be expected; the fewer objects in a scene, the less opportunity there is for participants to look at different things. We also found an effect of object animacy on fixation agreement (Figure 8); participants fixated animate objects (cats, dogs, cows) more consistently than inanimate objects (bottles, chairs, sofas). This may in part reflect an innate bias for faces to attract attention Banks and Ginsburg (1985). There was also a difference in participant agreement between the datasets. Mean agreement in fixation behavior was.88 (0.012 SEM) for the Pascal dataset but 0.85 (0.007 SEM) for the SUN09 dataset. This difference in fixation agreement likely reflects the difference in object content between these two datasets; Pascal images tend to depict a relatively small number of typically animate objects (a dog and cat sleeping on a sofa) whereas the SUN09 images tend to depict scenes of rooms containing many inanimate objects (a bedroom with lamps and end tables and chairs). Based on our observations and analyses, if one desires agreement in fixation behavior during scene viewing, one should use scenes depicting a small number of animate objects with clearly visible faces. This also has implications for scene understanding, suggesting that interpretations may be biased toward a small number of animate agents and the actions that they are performing.

### 3.3. What do people describe?

Descriptions are our measure of a person's scene understanding and reflect the relative importance placed on different content elements. To analyze the descriptions we first extracted the object words from the transcribed scene descriptions using a Part of Speech tagger (Phan, 2006) to tag the nouns. We then compared the extracted nouns to our selected object categories using WordNet distance (Wu and Palmer, 1994), keeping those nouns having a small distance (≤0.95). Because WordNet distance is not perfect, we supplemented this automated analysis with a manual inspection of the extracted word-object mappings to obtain our final sets of described nouns for each scene for analysis.

**Figure 9:**
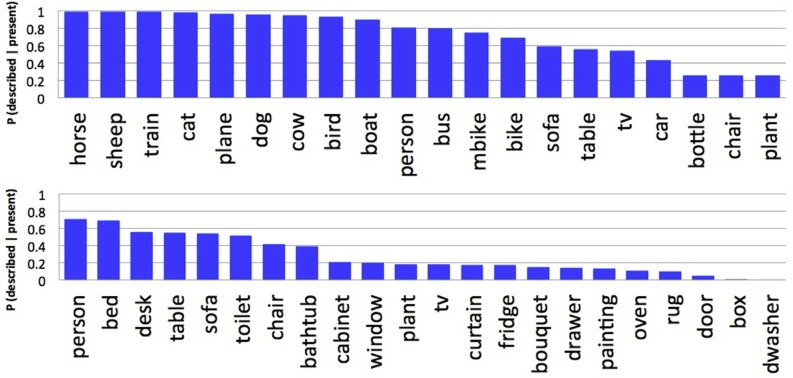
**Probability that an object category was described when present for the Pascal (top) and SUN09 (bottom) datasets**.

**Figure 10:**
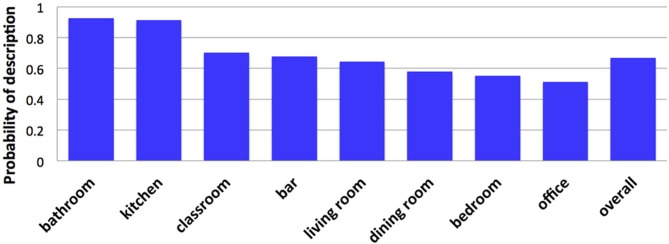
**Probability that a scene name was included in a description as a function of category**.

We found that people included in their descriptions 85.4 and 58.8% of the selected ground truth objects from the Pascal and SUN09 datasets, respectively. These differences are again likely due to the differing numbers of objects, and their animacy, between these datasets. Because there are more objects in the SUN09 scenes, people are less likely to describe all of the selected object categories. Previous work has also reported large differences between object categories in their probability of description (Berg et al., 2012). Paralleling our analyses of fixation preference and agreement, we found that animate objects were more likely to be described than inanimate objects. This is clearly illustrated in Figure 9, which orders the object categories based on their probability of description. For the Pascal dataset (top), animate objects such as horses and sheep were described with higher probability than inanimate objects such as chairs and potted plants. People were also more likely to be described in the SUN09 dataset (bottom), reflecting a similar trend, although the limited number of animate objects in this dataset makes the relationship less conclusive. No consistent description patterns were found among non-living objects capable of animacy, such as trains and cars and televisions. This suggests that the presence of faces, and not animacy, may be the more important factor in determining probability of description and fixation preference, and teasing apart these two possibilities will be an important direction for future research into scene understanding. Finally, we found that people often included a scene category name in their descriptions, and that the probability of this happening varied with the category of the scene (Figure 10). Determining the source of this variability, and whether fixation behavior can be used to predict scene naming, will be another important direction for future research.

### 3.4. What is the relationship between gaze and description?

There have been several studies exploring the relationship between object fixations and object descriptions [e.g., (Griffin and Bock, 2000), Meyer (2004), and Meyer et al. (2004)], but here we will focus on the two most fundamental: (1) whether people describe the objects they look at, and (2) whether people look at the objects they describe. We quantified these relationships by computing the probability that an object was fixated given that it was described, *P*(*fixated* | *described*) and the probability that an object would be described given that it was fixated, *P* (*described* | *fixated*). Note that we analyzed these probabilities under conditions in which the viewer and describer were the same person (SUN09) and when they were different people (Pascal). This latter scenario is interesting in that it speaks to the robustness of the relationships - that they might hold even across different individuals.

These results are shown in Table 2. We found a strong and clearly above chance relationship between gaze and description in the Pascal dataset (when the viewers and describers were different people)—the objects that were fixated affected the ones that were described, and vice versa. Potentially more interesting is the fact that these conditional probabilities were about equally high. We had expected the probability of fixating an object given that it was described to be higher than the probability of describing an object given that it was fixated. We thought this because, intuitively, one might fixate many more objects in a scene than they choose to describe, but to describe an object one might often need to fixate it. However, the Pascal dataset may have painted a distorted picture of these relationships, due to the fact that the scenes contained very few objects. Obviously, if every object was fixated and every object was described these probabilities would be 1. Indeed, when we conducted the same analyses for the SUN09 dataset we found a different, and more expected result. Subjects were far more likely to have fixated the objects that they described than to have described the objects that they fixated. This suggests that scene descriptions in our task were not simply a rote characterization of the fixated objects, but rather a higher-level interpretation assembled from the fixated objects that were deemed to be most important.

**Table 2:** **The fundamental relationships between object fixations and objects descriptions**.

	***P*(fixated|described)**	***P* (described|fixated)**
Pascal	86.56%	95.22%
SUN09	72.28%	21.98%

## 4. Gaze-enabled computer vision

In this section we discuss the potential for using human gaze as an input signal for two computer vision tasks—object detection and image annotation.

### 4.1. Analysis of human gaze with object detectors

**Figure 11:**
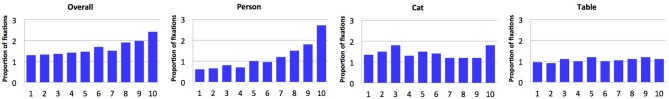
**Probability of fixation plotted as a function of object detector confidence score**. Scores are binned such that bin 10 indicates the top 10%, bin 9 indicates the top 10–20%, etc.

What are the correlations between the confidence of visual object detection systems and the objects that are fixated in a scene? Positive or negative correlations would provide insight into whether fixations have the potential to improve object detection performance. To evaluate this potential we obtained detection scores for the selected object categories from the Pascal dataset and plotted these as a function of fixation probability (Figure 11). We found that, averaged over category, participants tended to fixate on objects (in their bounding boxes) having higher detector confidence scores (*r* = 0.906). However, this varied by object category. As shown for the person category in Figure 11 (and also birds, boats, cars, cows, horses, sheep, and trains), there was a strong positive correlation between the probability of fixating these objects and their detection scores. For other categories, such as cats and tables, detection scores did not vary with their probability of fixation. This suggests that the value of integrating human gaze with object predictions from computer vision may depend on the object category.

**Figure 12:**
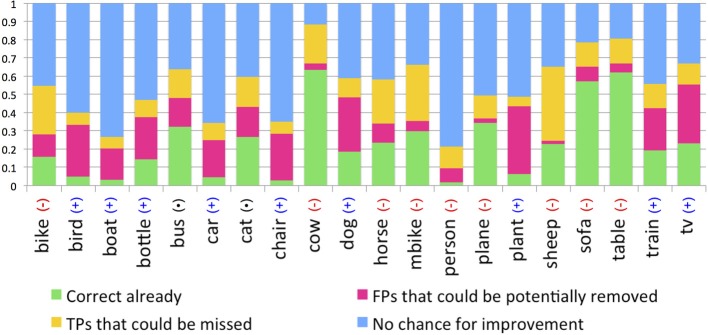
**Analyses indicating the potential for gaze to increase (pink), decrease (yellow), or to not affect (green and blue) the performance of object detectors**. For categories marked as (+) adding gaze would probably increase detection performance; for categories marked as (−) adding gaze would likely decrease performance.

Given that the value of human gaze inputs is category specific, we estimated the percentage of cases when fixations could be useful for object detection, when they would have no effect at all, and when they might actually be detrimental^1^. Specifically, we evaluated the potential for four scenarios. First, it is possible that no object detector box overlaps with the ground truth object (no true positive detection). For these cases, illustrated by the blue bars in Figure 12, gaze cannot be used to improve detection performance. A second possibility is that there are both true positive (TP) and false positive (FP) detection boxes overlapping with the object ground truth, and that there are more fixations falling in the FP box than in the TP box. In these cases biasing detection based on gaze would be expected to hurt object detection performance (yellow bars). A third possibility is that there would be more fixations in a TP box than in any other FP box, and for these cases gaze might improve object detection (pink bars). The green bars show cases in which the object detector already correctly predicted the object and no FP boxes overlapped with the ground truth. Under this fourth scenario, adding gaze would neither hurt nor help detection performance.

### 4.2. Object detection

The previous section estimated the potential object detection benefits that might result from a gaze-enabled object detection system; in this section we attempt to realize those benefits. We used deformable part models (Felzenszwalb et al., 2010) with their default thresholds to make detection predictions. We first considered the simplest possible method of using gaze—filter out all detections that do not contain any fixations within the bounding boxes (or, equivalently, to run object detectors only on those parts of the image containing fixations). Detection performance using this simple filtering algorithm is shown in Table 3 for the 20 Pascal categories. As predicted, many false positive detection boxes were removed, especially for lower-performing detectors such as bottles, chairs, plants, and people. However, it also removed many true positive detections for objects that were less likely to be fixated, such as bottles and plants. This resulted in improvements for some categories, but decreased detection performance overall. If the goal is to detect all of the objects in an image, even those ÒuninterestingÓ objects not usually fixated by people, then simple filtering by gaze is not advisable.

**Table 3:** **Average precision baseline, simple filtering, and gaze-enabled annotation prediction methods for the Pascal dataset**.

	**Bike**	**Bird**	**Boat**	**Bottle**	**Bus**	**Car**	**Cat**	**Chair**	**Cow**	**Dog**	
Baseline	61.7	38.2	44.1	27.9	55.0	50.8	42.9	30.3	66.6	65.7	**overall (mAP)**
Simple filtering	62.5	39.7	38.8	15.2	55.3	41.9	44.1	24.6	67.4	67.5	
Gaze-enabled	61.1	**40.9**	42.2	27.8	**55.5**	49.4	**47.1**	29.6	64.8	**66.3**	55.2
	**Horse**	**Mbike**	**Person**	**Plane**	**Plant**	**Sheep**	**Sofa**	**Table**	**Train**	**Tv**	52.3
Baseline	65.7	63.3	43.9	63.6	32.7	45.3	82.2	78.7	72.7	72.5	**55.3**
Simple filtering	63.8	60.2	40.6	63.6	16.6	38.5	82.6	79.3	73.9	70.4	
Gaze-enabled	**66.1**	63.1	43.6	60.4	**32.9**	45.0	**83.4**	78.5	**75.2**	**73.4**	

The values in bold indicate best performance by the gaze-enabled model.

We next tried a discriminative method in which classifiers were trained to distinguish between the true positive detections and the false positive detections output by the baseline detectors. Features used for classification included the baseline detection scores and features computed from gaze. To obtain the gaze features, we first created a fixation density map for each image (as described in Section 3.1), removed outliers by weighting these maps by fixation duration (Henderson, 2003), then averaged across participants to create an average fixation density map for each image. For each detection box we used as gaze features the average and maximum values on this fixation density map computed over the image patch described by the box. Classification was based on these three-dimensional feature vectors, consisting of the object detector score and the average and maximum values on the fixation density map.

The 1000 images from the Pascal dataset were split evenly into training and testing sets. Following convention, testing was evaluated using a standard 0.5 overlap required for true positives. However, for training we defined positive samples to be detection boxes having a lower overlap criterion (>0.30). We did this in order to obtain enough positive samples to train our classifier. For negative samples a stricter criterion was applied, overlap <0.01, and three iterations of hard-negative mining (Felzenszwalb et al., 2010) was used to iteratively add hard negative samples. Finally, we trained 20 classifiers, one per each of the selected object categories, using Support Vector Machines (SVMs) with an RBF Kernel and parameters set using 5-fold cross validation.

The results for this gaze-enabled detection are also provided in Table 3. Comparing these results to the baseline detectors and the simple filtering method, we see that gaze-enabled classifiers outperformed these other methods for many animal categories (e.g., birds, cats, dogs, and horses), trains and televisions, but performance was worse for planes, boats, cars, and cows. In general, we again found that gaze helped object detection for categories that are usually fixated, while it can hurt detection performance for those that are not (e.g., chairs). Additionally, we observed some cases where performance decreased due to detector confusion. For example, because the boat detector tended also to fire to planes, and given that people preferred to look at planes, gaze-enabled classification sometimes increased this confusion. To summarize, although overall detection performance (the mean of average precision across categories) was not greatly increased using gaze-enabled detection, we believe that this technique could be useful for detecting categories of objects that are consistently fixated.

### 4.3. Annotation prediction

**Figure 13:**
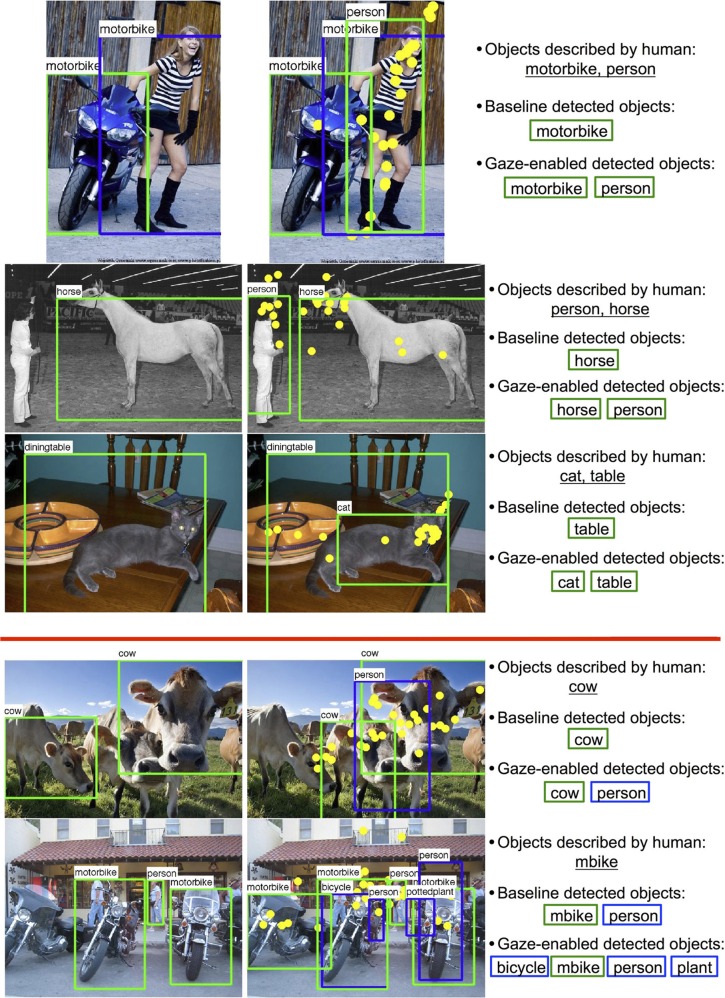
**Results of annotation prediction on the Pascal dataset. Left images:** Baseline detection, **Right images:** Gaze-enabled detection.

Image annotation, the outputting of a set of object tags that are descriptive of an image, is another application that might benefit from gaze cues. We obtained an annotation prediction by outputting the unique set of categories detected in an image, and considered a successful annotation to be one that matched the objects described by a person viewing the scene. Doing this for the simple filtering and gaze-enabled classification methods described in Section 4.2, we found gaze to be a slightly more useful cue for annotation than it was for object detection. Overall, both methods improved average annotation performance over baseline (Table 4), and were again especially helpful for those categories that where preferentially fixated and described (e.g., birds, cats, dogs, televisions). For other object categories, such as planes, bikes, and boats, we observed a decrease in annotation prediction, but this was often quite small. These benefits and costs can be broadly attributed to two sources: the removal of false positives (as shown for the televisions in Figure 2) and the gaze-enhanced potential for detector confusion (as for the detection of a person among the cows in Figure 13).

**Table 4:** **Average precision baseline, simple filtering, and gaze-enabled annotation prediction methods for the Pascal dataset**.

	**Bike**	**Bird**	**Boat**	**Bottle**	**Bus**	**Car**	**Cat**	**Chair**	**Cow**	**Dog**	
Baseline	75.8	42.6	57.1	49.3	74.9	71.4	44.8	49.2	84.9	66.2	
Simple filtering	76.8	44.8	51.9	51.8	75.1	76.1	46.1	48.6	85.4	67.9	**overall (mAP)**
Gaze-enabled	72.9	**47.2**	55.0	**49.5**	**75.2**	**72.7**	**49.1**	**50.3**	**85.2**	**67.9**	
	**Horse**	**Mbike**	**Person**	**Plane**	**Plant**	**Sheep**	**Sofa**	**Table**	**Train**	**Tv**	66.0
Baseline	85.9	81.9	64.5	67.6	39.8	63.3	73.0	76.3	82.9	68.7	66.8
Simple filtering	86.2	82.3	65.1	67.6	41.1	63.5	73.3	76.9	84.5	71.0	**66.9**
Gaze-enabled	**87.1**	**82.6**	**65.6**	66.4	38.6	**63.8**	72.9	76.3	**85.1**	**74.1**	

The values in bold indicate best performance by the gaze-enabled model.

## 5. Conclusions and future work

Scene understanding is more than just the knowledge of what objects appear in a scene, but equally true is that object detection plays an important role in scene understanding. In this exploratory study we examined the relationships between the content of a scene (the categories of objects appearing in an image), how the scene is viewed (the objects that people find most interesting), and how the scene is described (our measure of scene understanding). This exploration led to several findings: that people preferentially fixate animate objects having faces, that the locations of these fixations on objects can convey information about the object category, that people's agreement on which objects to fixate decreases with the number of objects in the scene but increases with the animacy of these objects, and that people are more likely to fixate the objects that they describe than to describe the objects that they fixate. Additionally, we found that fixation probability increases with object detector confidence for some categories, and we evaluated the potential for combining gaze and computer vision to improve the accuracy of object detection and annotation prediction. This effort, while revealing only modest benefits of adding gaze to standard object detectors, might inform future work on this topic by highlighting those cases in which gaze helped and hurt performance. Future work will build on this exploratory study by developing more intelligent and robust human-computer collaborative systems, and investigating more systematically the complex relationship between scene (and video) understanding and the object content of these images and how these objects and events are viewed.

### Conflict of interest statement

The authors declare that the research was conducted in the absence of any commercial or financial relationships that could be construed as a potential conflict of interest.

## Footnotes

^1^Note that we restricted this analysis to the Pascal dataset for our initial evaluation. Although the SUN09 dataset might have proved a more challenging test of our system, the reason for this would be uninteresting from our perspective– because object detectors trained for SUN09 showed generally poorer performance, poorer gaze-enabled object detection would be expected.

## References

[B1] Baldi P. F. Itti L. (2010). Of bits and wows: a bayesian theory of surprise with applications to attention. Neural Netw. 23, 649–666 10.1016/j.neunet.2009.12.007 20080025 PMC2860069

[B2] Banks M. S. Ginsburg A. P. (1985). Infant visual preferences: a review and new theoretical treatment, in ADV in Child Development and Behavior, Vol. 19, ed Reese H. W. (New York, NY: Academic Press), 207–246 10.1016/s0065-2407(08)60392-4 3911754

[B3] Berg A. C. Berg T. L. Daume H. Dodge J. Goyal A. Han X. (2012). Understanding and predicting importance in images, in IEEE Conference on Computer Vision and Pattern Recognition (CVPR) (Providence, RI), 3562–3569

[B4] Choi M. J. Lim J. Torralba A. Willsky A. (2010). Exploiting hierarchical context on a large database of object categories, in IEEE Conference on Computer VIsion and Pattern Recognition (CVPR) (San Francisco, CA), 129–136 10.1109/CVPR.2010.5540221

[B5] De Campos T. Csurka G. Perronnin F. (2012). Images as sets of locally weighted features. Comput. Vis. Image Und. 116, 68–85 10.1016/j.cviu.2011.07.011

[B6] Deng J. Berg A. Satheesh S. Su H. Khosla A. Li F.-F. (2012). Large Scale Visual Recognition Challenge. Available online at: http://www.image-net.org/challenges/LSVRC/2012/index

[B7] Deng J. Berg A. C. Li K. Fei-Fei L. (2010). What does classifying more than 10,000 image categories tell us?” in Proceedings of the 11th European Conference on Computer Vision (ECCV): Part V (Crete, Greece), 71–84 10.1007/978-3-642-15555-0_6

[B8] Deng J. Dong W. Socher R. Li L.-J. Li K. Fei-Fei L. (2009). ImageNet: a Large-Scale Hierarchical Image Database, in IEEE Conference on Computer VIsion and Pattern Recognition (CVPR) (Miami, FL), 248–255

[B9] Einhäuser W. Spain M. Perona P. (2008). Objects predict fixations better than early saliency. J. Vis. 8, 1–26 10.1167/8.14.18 19146319

[B10] Everingham M. Van Gool L. Williams C. K. I. Winn J. Zisserman A. (2008). The PASCAL Visual Object Classes Challenge 2008 (VOC2008) results. Available online at: http://www.pascal-network.org/challenges/VOC/voc2008/workshop/index.html

[B11] Everingham M. Van Gool L. Williams C. K. I. Winn J. Zisserman A. (2012). The PASCAL Visual Object Classes Challenge 2012 (VOC2012) results. Available online at: http://www.pascal-network.org/challenges/VOC/voc2012/workshop/index.html

[B12] Felzenszwalb P. Girshick R. McAllester D. Ramanan D. (2010). Object detection with discriminatively trained part-based models, IEEE Trans. Pattern Anal. Mach. Intell. 32, 1627–1645 10.1109/TPAMI.2009.167 20634557

[B13] Griffin Z. M. Bock K. (2000). What the eyes say about speaking. Psychol. Sci. 11, 24–279 10.1111/1467-9280.00255 11273384 PMC5536117

[B14] Henderson J. M. (2003). Human gaze control during real-world scene perception. Trends Cogn. Sci. 7, 498–504 10.1016/j.tics.2003.09.006 14585447

[B15] Itti L. (2005). Quantifying the contribution of low-level saliency to human eye movements in dynamic scenes. Vis. Cogn. 12, 1093–1123 10.1080/13506280444000661

[B16] Itti L. Baldi P. F. (2009). Bayesian surprise attracts human attention. Vis. Res. 49, 1295–1306 10.1016/j.visres.2008.09.007 18834898 PMC2782645

[B17] Itti L. Koch C. (2000). A saliency-based search mechanism for overt and covert shifts of visual attention. Vis. Res. 40, 1489–1506 10.1016/S0042-6989(99)00163-7 10788654

[B18] Itti L. Koch C. (2001). Computational modeling of visual attention. Nat. Rev. Neurosci. 2, 194–203 10.1038/35058500 11256080

[B19] Judd T. Durand F. Torralba A. (2011). Fixations on low-resolution images. J. Vis. 11, 1–20 10.1167/11.4.14 21518823

[B20] Judd T. Ehinger K. A. Durand F. Torralba A. (2009). Learning to predict where humans look, in International Conference on Computer Vision (ICCV) (Kyoto).

[B21] Krizhevsky A. Sutskever I. Hinton G. E. (2012). Imagenet classification with deep convolutional neural networks, in Conference on Advances in Neural Information Processing Systems 25 (Lake Tahoe, NV), 1106–1114

[B22] Lin Y. Lv F. Zhu S. Yang M. Cour T. Yu K. (2011). Large-scale image classification: fast feature extraction and svm training, in IEEE Conference on Computer VIsion and Pattern Recognition (CVPR) (Colorado Springs, CO), 1689–1696

[B23] Mackworth N. Morandi A. (1967). The gaze selects informative details within pictures. Percept. Psychophys. 2, 547–552 10.3758/BF03210264

[B24] Meyer A. van der Meulen F. Brooks A. (2004). Eye movements during speech planning: talking about present and remembered objects. Visual Cogn. 11, 553–576 10.1080/13506280344000248

[B25] Meyer A. S. (2004). The use of eye tracking in studies of sentence generation, in The Interface of Language, Vision, and Action: eye Movements and the Visual World, eds Henderson J. M. Ferreira F. (Hove: Psychology Press), 191–212

[B26] Neider M. B. Zelinsky G. J. (2006a). Scene context guides eye movements during search. Vis. Res. 46, 614–621 10.1016/j.visres.2005.08.025 16236336

[B27] Neider M. B. Zelinsky G. J. (2006b). Searching for camouflaged targets: effects of target-background similarity on visual search. Vis. Res. 46, 2217–2235 10.1016/j.visres.2006.01.006 16497351

[B28] Nuthmann A. Henderson J. M. (2010). Object-based attentional selection in scene viewing. J. Vis. 10, 1–19 10.1167/10.8.20 20884595

[B29] Pajak M. Nuthmann A. (2013). Object-based saccadic selection during scene perception: evidence from viewing position effects. J. Vis. 13, 1–21 10.1167/13.5.2 23547104

[B30] Parkhurst D. J. Law K. Niebur E. (2002). Modeling the role of salience in the allocation of overt visual selective attention. Vis. Res. 42, 107–123 10.1016/S0042-6989(01)00250-4 11804636

[B31] Perronnin F. Akata Z. Harchaoui Z. Schmid C. (2012). Towards good practice in large-scale learning for image classification, in IEEE Conference on Computer VIsion and Pattern Recognition (CVPR) (Providence, RI), 3482–3489 10.1109/TPAMI.2013.146 24457507

[B32] Phan X.-H. (2006). CRFTagger: CRF English POS Tagger. Available online at: http://crftagger.sourceforge.net/

[B33] Rashtchian C. Young P. Hodosh M. Hockenmaier J. (2010). Collecting image annotations using amazon's mechanical turk, in Proceedings of the NAACL HLT 2010 Workshop on Creating Speech and Language Data with Amazon's Mechanical Turk (Los Angeles, CA), 139–147

[B34] Renninger L. W. Vergheese P. Coughlan J. (2007). Where to look next? eye movements reduce local uncertainty. J. Vis. 3, 1–17 10.1167/7.3.6 17461684

[B35] Tatler B. W. (2007). The central fixation bias in scene viewing: Selecting an optimal viewing position independently of motor biases and image feature distributions. J. Vis. 7, 1–17 10.1167/7.14.4 18217799

[B36] Tatler B. W. Baddeley R. J. Vincent B. T. (2006). The long and the short of it: spatial statistics at fixation vary with saccade amplitude and task. Vis. Res. 46, 1857–1862 10.1016/j.visres.2005.12.005 16469349

[B37] Theeuwes J. (1994). Stimulus-driven capture and attentional set: selective search for color and visual abrupt onsets. J. Exp. Psychol. 20, 799–806 8083635 10.1037//0096-1523.20.4.799

[B38] Theeuwes J. Kramer A. Hahn S. Irwin D. Zelinsky G. (1999). Influence of attentional capture on oculomotor control. J. Exp. Psychol. 25, 1595–1608 10641312 10.1037//0096-1523.25.6.1595

[B39] Wu Z. Palmer M. (1994). Verbs semantics and lexical selection, in Proceedings of the 32nd Annual Meeting on Association for Computational Linguistics (Las Cruces, NM), 133–138 10.3115/981732.981751

[B40] Yarbus A. (1967). Eye Movements and Vision. (New York, NY: Plenum Press), 10.1007/978-1-4899-5379-7

[B41] Zelinsky G. J. (2008). A theory of eye movements during target acquisition. Psychol. Rev. 115, 787–835 10.1037/a0013118 18954205 PMC2577318

[B42] Zelinsky G. J. Schmidt J. (2009). An effect of referential scene constraint on search implies scene segmentation. Vis. Cogn. 17, 1004–1028 10.1080/13506280902764315

